# Supplementary material for: Predicting visual function by interpreting a neuronal wiring diagram
Source: Nature. 2024 Oct 2;634(8032):113–23. doi: 10.1038/s41586-024-07953-5 (PMC11446822; doi:10.1038/s41586-024-07953-5)
Supplement: Supplementary file 6 — CRF and ERF predictions for individual TmY4 and TmY9 cells. Analogous to Supplementary Data 3, but for TmY target types. Shown are the top four monosynaptic pathways, the strongest pathway passing through each of the top ten intermediary types (ranking from Extended Data Fig. 7), and the trisynaptic pathway Tm1–TmY–Dm3–TmY (see the section entitled Prediction of spatial normalization). [file 41586_2024_7953_MOESM6_ESM.zip › DataS4/TmY9qΓèÑ/720575940634756889.pdf]

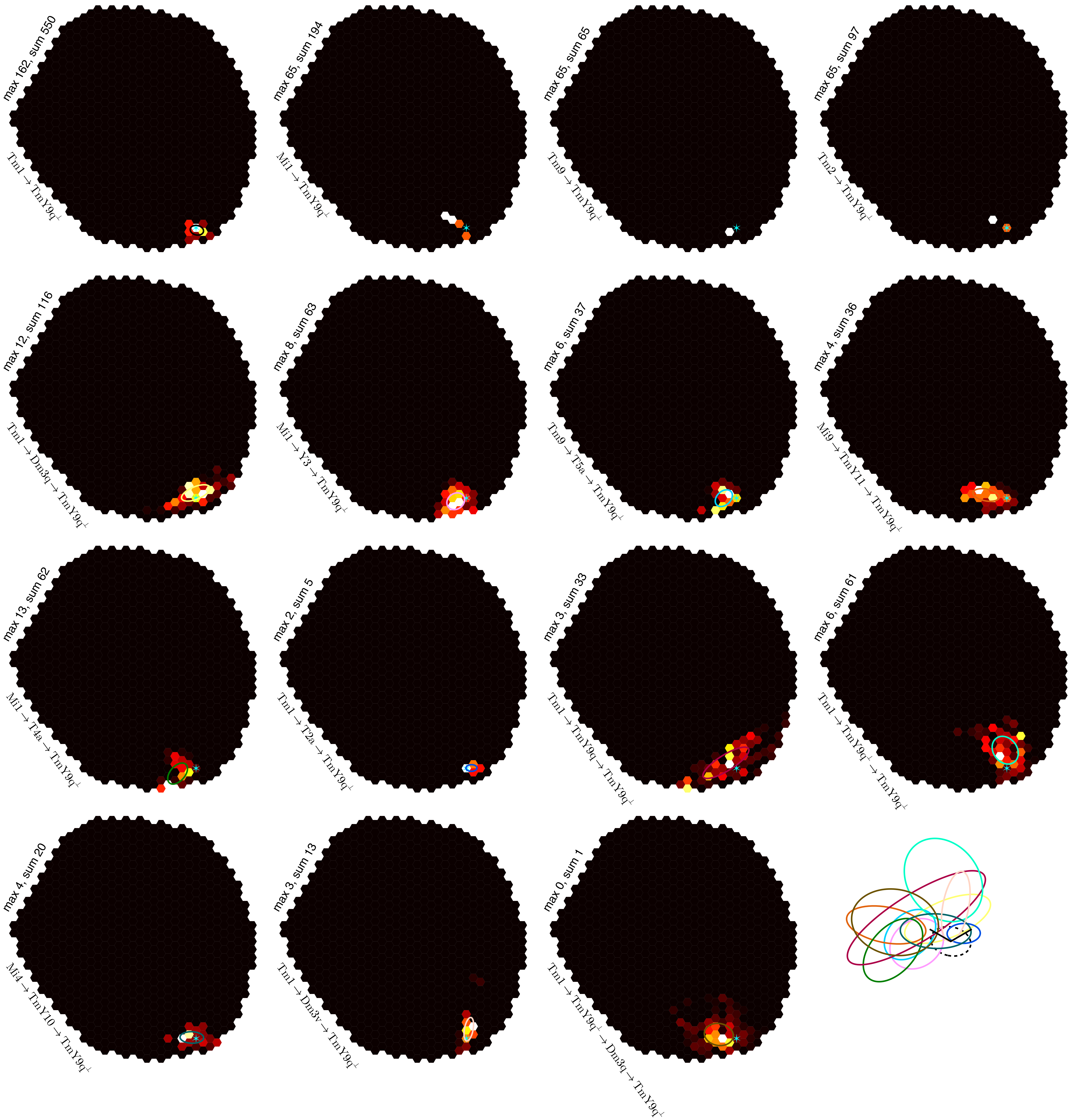
max 162, sum 550
Tm1 → TmY9q⊥
max 65, sum 194
Mi1 → TmY9q⊥
max 65, sum 65
Tm9 → TmY9q⊥
max 65, sum 97
Tm2 → TmY9q⊥
max 12, sum 116
Tm1 → Dm3q → TmY9q⊥
max 8, sum 63
Mi1 → Y3 → TmY9q⊥
max 6, sum 37
Tm9 → T5a → TmY9q⊥
max 4, sum 36
Mi9 → TmY11 → TmY9q⊥
max 13, sum 62
Mi1 → T4a → TmY9q⊥
max 2, sum 5
Tm1 → T2a → TmY9q⊥
max 3, sum 33
Tm1 → TmY9q → TmY9q⊥
max 6, sum 61
Tm1 → TmY9q⊥ → TmY9q⊥
max 4, sum 20
Mi4 → TmY10 → TmY9q⊥
max 3, sum 13
Tm1 → Dm3v → TmY9q⊥
max 0, sum 1
Tm1 → TmY9q⊥ → Dm3q → TmY9q⊥
